# Cannabis Use and Endocannabinoid Receptor Genes: A Pilot Study on Their Interaction on Brain Activity in First-Episode Psychosis

**DOI:** 10.3390/ijms24087501

**Published:** 2023-04-19

**Authors:** Maitane Oscoz-Irurozqui, Carmen Almodóvar-Payá, Maria Guardiola-Ripoll, Amalia Guerrero-Pedraza, Noemí Hostalet, Raymond Salvador, Maria Isabel Carrión, Teresa Maristany, Edith Pomarol-Clotet, Mar Fatjó-Vilas

**Affiliations:** 1 FIDMAG Germanes Hospitalàries Research Foundation, Av Jordà 8, 08035 Barcelona, Spain; moscoz@alumni.unav.es (M.O.-I.); calmodovar@fidmag.org (C.A.-P.); mguardiola@fidmag.org (M.G.-R.); aguerrero.hbmenni@hospitalarias.es (A.G.-P.); nhostalet@fidmag.org (N.H.); rsalvador@fidmag.org (R.S.); 2 Centro de Salud Mental Errenteria-Osakidetza, Av Galtzaraborda 69-75, 20100 Errenteria, Guipúzcoa, Spain; 3 Centro de Investigación Biomédica en Red de Salud Mental (CIBERSAM), Instituto de Salud Carlos III, Av Monforte de Lemos, 3-5, Pabellón 11, Planta 0, 28029 Madrid, Spain; 4 Hospital Benito Menni CASM, C/Doctor Antoni Pujadas 38, 08830 Sant Boi de Llobregat, Barcelona, Spain; 5 Hospital Sant Rafael, Passeig de la Vall d’Hebron, 107, 08035 Barcelona, Spain; micarrion.hsrafael@hospitalarias.es; 6 Diagnostic Imaging Department, Hospital Sant Joan de Déu Research Foundation, Passeig de Sant Joan de Déu, 2, 08950 Esplugues de Llobregat, Barcelona, Spain; tmaristany@sjdhospitalbarcelona.org; 7 Departament de Biologia Evolutiva, Ecologia i Ciències Ambientals, Facultat de Biologia, Universitat de Barcelona, Avinguda Diagonal, 643, 08028 Barcelona, Spain

**Keywords:** first-episode of psychosis, cannabis use, cannabinoid receptor genes, fMRI working memory

## Abstract

The role of both cannabis use and genetic background has been shown in the risk for psychosis. However, the effect of the interplay between cannabis and variability at the endocannabinoid receptor genes on the neurobiological underpinnings of psychosis remains inconclusive. Through a case-only design, including patients with a first-episode of psychosis (n = 40) classified as cannabis users (50%) and non-users (50%), we aimed to evaluate the interaction between cannabis use and common genetic variants at the endocannabinoid receptor genes on brain activity. Genetic variability was assessed by genotyping two Single Nucleotide Polymorphisms (SNP) at the cannabinoid receptor type 1 gene (*CNR1*; rs1049353) and cannabinoid receptor type 2 gene *(CNR2*; rs2501431). Functional Magnetic Resonance Imaging (fMRI) data were obtained while performing the n-back task. Gene × cannabis interaction models evidenced a combined effect of *CNR1* and *CNR2* genotypes and cannabis use on brain activity in different brain areas, such as the caudate nucleus, the cingulate cortex and the orbitofrontal cortex. These findings suggest a joint role of cannabis use and cannabinoid receptor genetic background on brain function in first-episode psychosis, possibly through the impact on brain areas relevant to the reward circuit.

## 1. Introduction

A complex interplay between genetic and environmental factors is involved in the aetiology of psychosis. Both quantitative and molecular studies have well-established the heritability of psychotic disorders [1,2,3]; however, genome-wide association data have shown that the common genetic variability explains only a part of the variance in the vulnerability [4,5]. This reflects that environmental exposures may account for a proportion of the liability. Indeed, epidemiological studies have identified many environmental risk factors associated with psychosis, such as obstetric and perinatal complications, early infections, substance abuse, urbanicity and migration or psychosocial stress [6,7,8]. Among them, cannabis use has captured much attention since several population-based studies consistently reported that cannabis consumptionis associated with an increased risk for psychotic experiences [9] and psychotic disorders [10,11]. Additionally, studies based on clinical samples have described a higher prevalence of cannabis use in subjects with a first-episode of psychosis as compared to the general population [12,13,14] and that the age at which cannabis use begins appears to correlate with the age at onset of psychosis [15,16]. Despite this knowledge, the nature of the relationship between genetic liability, cannabis use and psychosis development is not fully understood.

The association between cannabis use and psychosis is mediated by the effect of exogenous cannabinoids on the endocannabinoid system (ECBS), which is intimately related to the dopamine system [16]. Concretely, the ∆9-tetrahydrocannabinol (THC), the main psychoactive component of cannabis, is a partial agonist of the cannabinoid G protein-coupled receptors (cannabinoid receptor type 1 (CB1R) and type 2 (CB2R)) and binds to them with strong affinity.

CB1R is the most abundant receptor in the mammalian brain; and it is expressed in the presynaptic terminals in the basal ganglia, the cerebellum, the hippocampus, the cingulate, the amygdala, and the prefrontal cortex [17]. It is localised on the gamma-aminobutyric acid (GABA-ergic) and glutamatergic neurons, acting as a retrograde feedback system to modulate dopamine transmissions through inputs to dopaminergic neurons in the ventral midbrain that express these receptors [18,19].

CB2R was first detected in the periphery but later in the brain [20], particularly in the amygdala, hippocampus, cerebellum, nucleus accumbens, caudate/putamen and cortex [21]. Also, recent advances indicate that CB2R is expressed in both the brain’s neurons and glial cells [22]. Given that CB2R appears to be mainly postsynaptic, it is thought that the activation of CB2R may play the opposite effect of CB1R [23].

The role of CB1R and CB2R signalling pathways in the modulation of various dopamine-associated behaviours [18,19,24] and their inducibility by external factors have emphasised their critical role in multiple cellular and behavioural functions, involving both cognitive and psychiatric conditions, such as substance abuse [23].

The cortico-basal ganglia-thalamic areas that densely express cannabinoid receptors are part of a broader network subserving working memory functions [25]. Alterations in this cognitive dimension are consistently reported in patients with psychotic disorders [26,27]. In this line, a recent meta-analysis found that working memory is one of the most prominently affected cognitive domains by acute administration of partial CB1R agonists [28]. Nevertheless, current data offer mixed results with regard to the direction of the effect of cannabis on working memory performance. Some studies describe a worse cognitive performance associated with cannabis use both in healthy subjects [29,30] and individuals with a psychotic disorder [31,32]; others show better performance in patients using cannabis [33,34], and there are also studies showing no effect [35,36].

From functional Magnetic Resonance Imaging (fMRI), most studies based on healthy cannabis users (CU) report changes in the activation patterns associated with the performance of working memory and attention tasks [36,37,38,39]. However, the conclusions of available studies are markedly inconsistent and hardly comparable because of various confounders. Regarding the fMRI studies exploring the effect of cannabis use on cognitive functioning in subjects with psychosis, the evidence is scarce and based only on three previous studies with limited sample sizes of patients with chronic schizophrenia. These previous fMRI studies report more preserved cognitive functioning in CU than cannabis non-users (CNU) [40,41,42]. This same pattern was observed in studies evaluating neuroanatomical or neurocognitive differences between patients with psychotic disorders exposed to cannabis and patients not exposed to it [33,34,43]. However, to our knowledge, no fMRI study has ever been focused on exploring the cannabis use effect in patients with a first-episode of psychosis.

The fact that only a small proportion of CU develop psychosis, and the observed heterogeneity of cannabis effects, suggest that genetic variability on ECBS-related genes could be involved in the differential sensitivity to cannabis use effects between individuals [44,45].

In this sense, some studies have associated the gene encoding for the CB1R (*CNR1* gene) with the risk for schizophrenia [46,47], with the performance in various cognitive dimensions, such as executive function, attention or memory [48,49], and with changes in brain volumes [48,50]. However, other inconclusive results [51] stress the need for further research on the role of endocannabinoid genetics in schizophrenia and its associated phenotypes. Regarding brain imaging phenotypes, some studies report that cannabis use, in the context of specific *CNR1* genotypes, may contribute to brain volume differences, both in patients with schizophrenia and healthy volunteers [48,50,52,53]. Concerning fMRI approaches, the existing studies are only based on healthy individuals and report the interplay between *CNR1* and cannabis use on prefrontal activity, connectivity, and behavioural performance during the n-back task [54,55]. In addition, a polygenic approach, based on the co-expression network of *CNR1*, highlighted the interaction between cannabis use and the *CNR1*-network polygenetic score on the dorsolateral prefrontal activity during working memory processing in healthy subjects [56]. Such results suggest that cannabis use affects the physiological relationship between genetically induced expression of *CNR1* and prefrontal working memory processing. However, as far as we know, no previous fMRI studies have analysed the interplay of the endocannabinoid genetic variants and cannabis use in patients with psychosis. Similarly, research is scarce on the role of polymorphic variability at the gene encoding for the CB2R (*CNR2* gene) in the risk for psychosis [57] and, to our knowledge, no studies have assessed the relation between *CNR2* and cannabis use through fMRI, neither in healthy subjects nor in patients with schizophrenia.

### Aims of the Study

This background highlighs the need for further research on the effect of cannabis use on the emergence of the first-episode of psychosis. In particular, as genetic factors have been shown to influence cannabis association with psychosis outcomes, we aimed to analyse the role of common genetic variability at cannabinoid receptor genes in mediating the effect of cannabis use on brain activity. Therefore, we have conducted a Gene × Environment study to assess the role of two common genetic variants at *CNR1* (rs1049353) and *CNR2* (rs2501431) genes and cannabis use on working memory brain function in a case-only sample of patients with a first-episode of psychosis. All participants underwent an fMRI protocol, including a working memory task (n-back), which allowed the brain activity comparison between CU and CNU conditional to the *CNR1* and *CNR2* genotypes.

## 2. Results

### 2.1. Sample Description

Out of 40 participants in the study, 20 (50%) were classified as CU and the rest as CNU. There were no significant differences in the demographic, clinical, and mean dose of antipsychotic treatment data between the two groups (Table 1).

The genotypic distribution of the rs1049353 and rs2501431 polymorphisms is shown in Table 2. Minor allele frequencies were T = 0.2 and G = 0.38, respectively.

### 2.2. Neuroimaging Association Analyses

There were no differences on any fMRI contrast between CU and CNU.

#### 2.2.1. Cannabis Use × *CNR1* Variability Interaction Effect on Brain Activity

**Figure 1:**
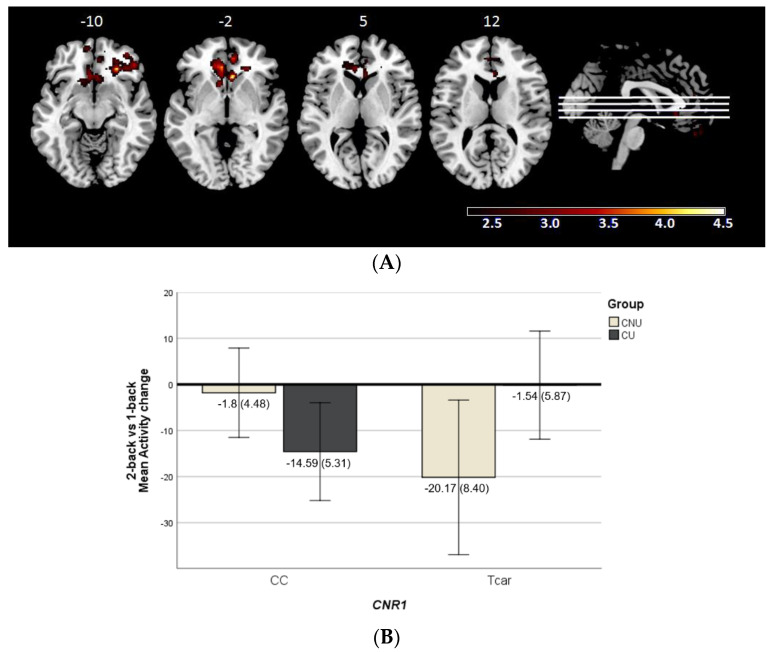
(**A**) Brain regions included in the cluster showing significant cannabinoid receptor type 1 gene (*CNR1*, rs1049353) × cannabis use interaction (CNU—cannabis non-users; CU—cannabis users) in the 2-back vs. 1-back contrast. The right side of the image represents the right side of the brain. Montreal Neurological Institute (MNI) coordinates are given for the shown slice. Units of the bar are β values from the regression model standardised to Z scores. (**B**) Bar plot showing marginal means of the mean activity change values (±2SE) for the significant cluster where *CNR1* × cannabis use interaction is detected in the 2-back vs. 1-back contrast.

The gene × environment interaction analyses in the 2-back vs. 1-back contrast revealed a significant effect of *CNR1* × cannabis encompassing one cluster located within the right medial and superior temporal pole and bilaterally within the orbitofrontal cortex, anterior cingulate cortex and caudate (2620 voxels, peak activation at MNI (Montreal Neurological Institute coordinates) [48, 18, −28], z = 4.4, and *p* = 2.98 × 10^−7^) (Figure 1A).

To better understand the directionality of the results, we extracted the corresponding mean activity scores within the cluster. It must be acknowledged that the obtained scores represent the mean activity change occurred between the two n-back task levels (the 1-back and the 2-back). As seen in Figure 1B, CU with the CC genotype and CNU carrying the T allele showed a functional modulation in response to the higher cognitive demand associated with the 2-back, while the others did not almost change their activity. This represents an inverse genotype-related pattern between CU and CNU.

This cluster showed a complete overlap with the one identified in the 1-back vs. baseline contrast (835 voxels, peak activation at MNI [8, 52, −12], z = 3.9, and *p* = 0.0093); while in the 2-back vs. baseline, no interaction was found.

#### 2.2.2. Cannabis Use × *CNR2* Variability Interaction Effect on Brain Function

**Figure 2:**
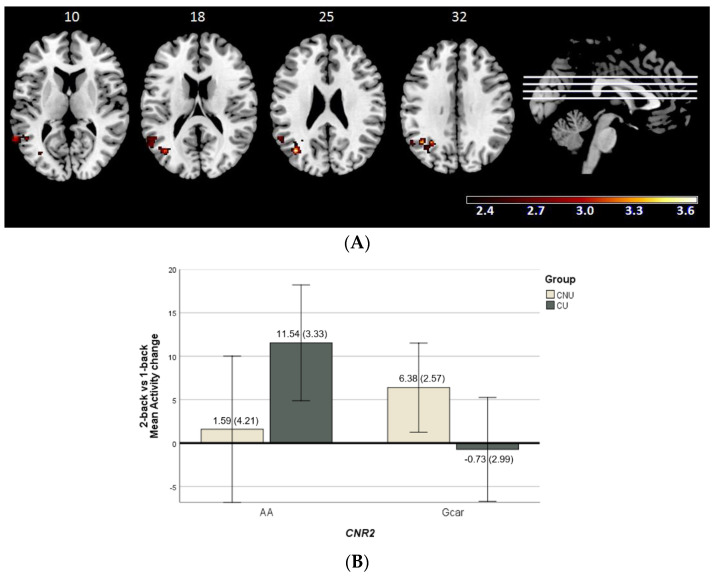
(**A**) Brain regions included in the cluster showing significant cannabinoid receptor type 2 gene (*CNR2*, rs2501431) × cannabis use interaction (CNU–cannabis non-users; CU–cannabis users) in the 2-back vs. 1-back contrast. The right side of the image represents the right side of the brain. Montreal Neurological Institute (MNI) coordinates are given for the shown slice. Units of the bar are β values from the regression model standardised to Z scores. (**B**) Bar plot showing marginal means of the mean activity change values (± 2SE) for the significant cluster where *CNR2* × cannabis use interaction is detected in the 2-back vs. 1-back contrast.

Cannabis use and the *CNR2* polymorphism showed a significant interplay involving one cluster in the 2-back vs. 1-back contrast (792 voxels, peak activation at MNI [−38, −68, 48], z = 3.65, and *p* = 0.00955). The cluster was located at the left hemisphere, at the middle temporal gyrus, middle occipital gyrus, supramarginal gyrus and angular gyrus (Figure 2A). With regards to the mean activity scores in this cluster, there was an opposite genotype-related pattern between CU and CNU (Figure 2B).

No interaction effects were found in the other two contrasts (2-back or 1-back vs. baseline).

### 2.3. N-Back Behavioural Performance Analyses

**Figure 3:**
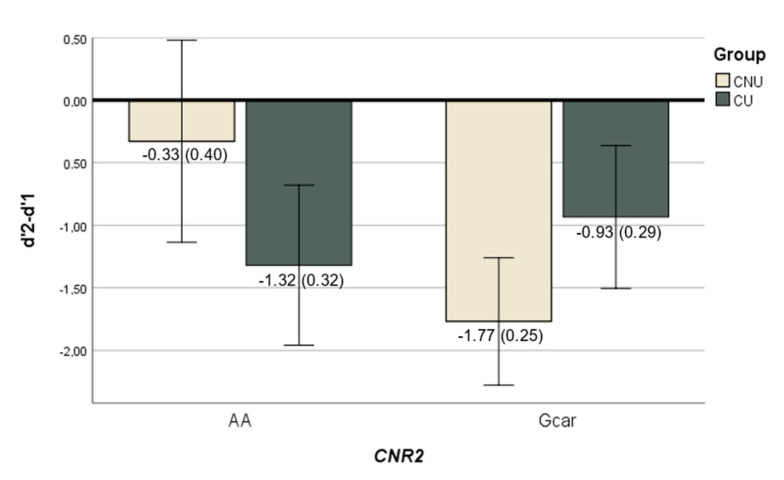
Bar plot showing the significant interaction between *CNR2 CNR2* (rs2501431) × cannabis use (CNU—cannabis non-users; CU—cannabis users) in the n-back task performance. Each bar represents the marginal mean of the d′2-d′1 score (±2SE) (which measures the behavioural response to the increased difficulty of the task), separately by genotypes and cannabis use groups.

Linear regression revealed no effect of cannabis use on n-back performance in any difficulty level of the task (d′1, d′2 and d′2-d′1). Furthermore, no significant interaction was found between *CNR1* and cannabis use on them. With regards to the interplay between cannabis use and *CNR2*, we found an interaction effect for *CNR2* and cannabis use on d′1 (ß = −1.58, *p* = 0.035) and d′2-d′1 (ß = 1.82, *p* = 0.012) levels (Figure 3); but not on d′2.

## 3. Discussion

As far as we know, this is the first study to examine the interplay of cannabis use and the endocannabinoid receptor genes on functional neuroimaging-derived phenotypes in patients with a first-episode of psychosis. We did not detect an effect of cannabis use on brain activity when performing the n-back task. Instead, our data indicate that genetic modulation has an impact on the effect of cannabis use on brain response.

Regarding the analysis of the main effect of cannabis use on the n-back task-related brain activity, we did not observe differences between CU and CNU. These findings diverge from previous fMRI studies on schizophrenia, which showed higher activity rates in CU as compared to CNU in multiple brain areas associated with different cognitive dimensions (executive functions, verbal processing and attention, emotional processing and visuospatial abilities) [40,41,42]. However, our results should be interpreted in light of several aspects.

First, these previous findings were based on different tasks (e.g., emotional memory task, mental rotation task, cognitive task-present and task-absent conditions). Second, the design based on first-episode psychosis cases limits the main cannabis use effect on brain function before the onset of the psychosis, while schizophrenia-based samples (such as in previous fMRI studies) do not exclude the impact of cannabis use after the illness onset. Third, these previous studies include patients with an illness duration longer than ten years, which hampers the direct comparison of the result because duration-related illness factors can also affect brain activity. Fourth, general population-based fMRI studies performed with CU in the non-intoxicated state predominantly show the opposite effects to those observed in chronic patients with schizophrenia. Thus, CU show attenuated brain activity or activation of compensatory regions compared to CNU (for review, see [57]). In contrast, the scarce fMRI literature about patients diagnosed with schizophrenia suggests that CUtend to show better preserved functioning in areas associated with the task than non-users [40,41,42].

Concerning our cannabis × gene approach, our data seem to support the idea that the individual genetic background of the ECBS may influence the effect of cannabis on the brain’s functional response to specific cognitive demands, which could be linked to the participation of this system in the vulnerability for psychosis [44,45]. Notably, we found evidence of a genotype × cannabis use interaction for both endocannabinoid receptor genes (*CNR1* and *CNR2*) in the 2-back vs. 1-back contrast, suggesting a modulation effect of genetics on neuronal dynamics related to working memory after cannabis exposure.

For *CNR1*, we observed an interplay between rs1049353 and cannabis use in areas that are known to have reward-related functions [58,59]. In line with our results, the ECBS has been implicated in reward-processing and reward-seeking behaviour (reviewed in [60]), which is also supported by the fact that CB1R is densely expressed in areas associated with reward processing (e.g., hippocampus, amygdala, prefrontal cortex, anterior cingulate, striatum or VTA). In this sense, substance abuse is associated with increased activity of dopaminergic neurons, which, in turn, release endocannabinoids through the activation of CB1Rs in GABA or glutamate-containing neurons [18]. Then, alterations in the ECBS by external ligands could lead to dysregulation in their associated neurotransmitter systems and functions.

Regarding the *CNR2* gene, our findings also suggest that the polymorphism rs25014131 and cannabis use are associated with brain activity changes in patients with a first-episode psychosis. While brain CB2R has lower expression levels than CB1R [20], its expression appears to be altered under certain pathological conditions (e.g., addiction, inflammation, and anxiety), suggesting that these receptors are highly inducible (reviewed in [22]). In this regard, Ishiguro et al. [61] described an increased risk of schizophrenia among people with low CB2R function, measured through its expression. These authors also identified functional changes in CB2R attending to nucleotide variants. Thus, according to these data [61], it could be hypothesised that, in patients with psychotic disorders, *CNR2* genetic variability influences CB2R activity and, finally, brain activity due to the regulation role of ECBS on other neural systems. Interestingly, we found opposite brain activation patterns for *CNR1* and *CNR2* according to cannabis use. While further data are needed, this result could be interpreted in line with the described possible opposite roles of CB1R and CB2R [23] in the reinforcement processes of substance abuse and dopamine-related behaviours.

Finally, when looking at the task execution, aligned with Løberg et al. [34] or Potvin et al. [40], we did not find differences in behavioural performance between CU and CNU. However, regarding the interaction effect between genetic variability and cannabis use on task performance, we report a significant impact of *CNR2*, but not of *CNR1*. Task performance in CU was similar for both genotypic *CNR2* variants, while among the CNU, the genotype seems to play a differential modulating role. Interestingly, when analysing together the functional and behavioural data, we observe that the genotypic groups showing a larger modulatory effect in response to the increasing task difficulty also show a wider task performance drop between the 2-back vs. the 1-back task (Gcar -for CNU- and AA -for CU).

Our study should be interpreted in the context of some limitations. First, we identified cannabis use from self-reports and medical records, and we used cannabis use as a dichotomised no/yes category. While this classification has also been implemented in previous studies [34,42,50], more extensive cannabis intake data (% of THC, age at onset, frequency, consumption via, etc.) will be valuable in future studies. Second, the sample size of our study limits our conclusions, and that is why it should be considered as a pilot approach that warrants further research. However, our sample based on the first-episode of psychosis contrasts with the previous fMRI and cannabis use studies to date [40,41,42], based on patients with chronic schizophrenia. To avoid potential confounding effects of differences associated with cannabis use before and after the onset of the psychotic symptoms, studies focused on the first-episode are especially useful. In addition, while we are aware of the imbalanced sample size regarding gender (77.5% males) as recent studies have proven sex-dependent effects of cannabis [62] and the ECBS [63], from our sample size, we could not develop sex-specific analyses. In this sense, our analyses of the whole sample must be compared with previous studies using mixed samples and also showing a predominance of males [42,50]. Third, the unavailability of a control group restricts interpretations of the role of cannabis use and genetic make-up to early phases of psychotic disorders and precludes analyses in terms of health–disease status and vulnerability. Finally, to improve our understanding of causal interactions between relevant factors for psychosis, longitudinal designs would be more appropriate. For instance, future studies would benefit from follow-up studies of high-risk individuals and the analysis of cannabis use jointly with the genetic profiling of different endocannabinoid system components [45]. Additionally, based on recent data showing different epigenetic factors contributing to the regulation of cannabinoid receptors [64,65] and epigenetic changes linked to the conversion to psychosis [66], the combined analysis of genetic and epigenetic variants appears as a promising avenue for novel therapeutic targets and innovative treatment strategies.

## 4. Materials and Methods

### 4.1. Participants and Study Design

The sample comprised 40 patients with a first-episode of non-affective psychosis recruited from a psychiatric hospital in the area of Barcelona. They were all experiencing their first onset of psychosis, and the duration of psychotic symptoms was <18 months. All of them were adults (age ≥ 18 years), of European origin and right-handed.

Exclusion criteria were: (i) age above 65 years, (ii) premorbid Intelligence Quotient (IQ) < 75, (iii) history of brain trauma with loss of consciousness or neurological condition, (iv) presence of a DSM-IV affective psychotic diagnosis (mania, hypomania, and major depression with psychotic symptoms).

The patients underwent a diagnostic evaluation at admission using the Spanish version of the Structured Clinical Interview for DSM-IV (SCID). The distribution of the diagnoses was as follows: schizophrenia (n = 22), schizoaffective disorder (n = 3), delusional disorder (n = 1) and unspecified psychosis (n = 14). Symptoms were scored using the patients’ clinical evaluation, which included the Positive and Negative Symptoms Scale (PANSS) [67]. Based on the PANSS, Positive, Negative and Disorganised Syndrome scores were calculated [68]. Premorbid IQ was estimated using the Word Accentuation Test (Test de Acentuación de Palabras, TAP [69]). Diagnostic evaluation and clinical and neuropsychological assessments were carried out by an experienced psychiatrist and psychologist, respectively.

### 4.2. Cannabis Use 

All participants were asked about their use of illicit drugs, and a review of their medical history (both electronic and paper records) was carried out to check the information. Patients with alcohol/substance (except cannabis) abuse or dependence within six months before participation were excluded.

Cannabis use was assessed over each participant’s lifetime. Cannabis non-users (CNU) were those that never used cannabis or used it only once. Cannabis users (CU) were those with regular cannabis use, and the majority of these patients (85%) met the criteria for the Diagnostic and Statistical Manual of Mental Disorders (DSM-IV-TR) of cannabis abuse or dependence. When they underwent fMRI and carried out the other assessments, they had been abstinent for at least one week because of hospital admission.

### 4.3. fMRI Data Acquisition and fMRI Task Description

#### 4.3.1. Acquisition Parameters

For each individual, 266 volumes were acquired during a scanning session from a 1.5-T GE Sigma scanner (General Electric Medical Systems, Milwaukee, WI, USA). A gradient echo-planar imaging (EPI) sequence, depicting the blood oxygenation level-dependent (BOLD) contrast was used. Each volume contained 16 axial planes acquired with the following parameters: repetition time (TR) = 2000 ms, echo time (TE) = 20 ms, flip angle = 70°, section thickness = 7 mm, section skip = 0.7 mm, and in-plane resolution = 3 × 3 mm. To avert T1 saturation effects, the 10 initial volumes were removed.

#### 4.3.2. N-Back Task

All subjects completed a sequential letter version of the n-back task [70] during the fMRI protocol. The task execution engages many storages and executive processes related to working memory and attention. The task has two levels of memory load (1-back and 2-back) presented in a blocked design manner. Each block consists of 24 letters that are shown every 2 s (1 s on and 1 s off), and all blocks contain five repetitions located randomly within the blocks. Individuals had to indicate repetitions by pressing a button. Four 1-back and four 2-back blocks were presented in an interleaved way, and between them, a baseline stimulus (an asterisk flashing with the same frequency as the letters) was presented for 16 s. Characters were shown in green for 1-back blocks and in red for 2-back blocks to identify which level had to be performed. All participants went through a training session outside the scanner the same day and before the scanning session.

#### 4.3.3. N-Back Performance

The behavioural measure used was the signal detection theory index sensitivity, d′ score [71]. Higher values of d′ indicate a better ability to discriminate between targets and distractors, while negative values indicate that subjects were not performing the task. Therefore, those individuals with negative d′ values (d′1 for 1-back level and d′2 for 2-back level) in any of the two difficulty levels of the task were not included in any further analyses.

Following the same procedure as Egli et al. [72], we used the difference in performances, named d′2-d′1 score, as a measure to evaluate the behavioural response to the increased difficulty of the task. Smaller values of the d′2-d′1 score indicate a lesser ability to respond to increasing cognitive demand.

### 4.4. Genotyping

Genomic DNA was extracted for all individuals either from buccal mucosa using cotton swabs and ATP Genomic DNA Mini Kit Tissue (Teknokroma Analítica, S.A., Sant Cugat del Vallès, Barcelona, Spain) or from peripheral blood cells using Realpure SSS Kit for DNA Extraction (Durviz, S.L.U, Valencia, Spain). Two single nucleotide polymorphisms (SNP) were genotyped: rs1049353 at *CNR1* (Chr: 6q14-q15) and rs2501431 at *CNR2* (Chr: 1p34-p35) genes. These SNPs were selected based on the following: (i) previous studies on their association with schizophrenia or cannabis use [50,73,74,75,76], and (ii) MAF in the European population > 10%. Genotyping was conducted using a fluorescence-based allelic discrimination procedure (Applied Biosystems TaqMan 5′-exonuclease assays) using standard conditions. The genotyping call rate was 93.02% for both SNPs. The method’s accuracy was tested by re-genotyping 10% of the samples and confirming all the repeated genotypes. Genotype frequencies were in Hardy–Weinberg equilibrium.

### 4.5. Statistical Analyses

#### 4.5.1. Clinical and Demographics Data Analyses

Clinical and demographic data of CUand CNU were evaluated through *t*-student and chi-square tests using SPSS 23.0 software (IBM SPSS Statistics for Windows, version 23.0, released 2015, IBM Corporation, Armonk, NY, USA).

#### 4.5.2. Neuroimaging Association Analyses

The fMRI image analyses were performed with the FEAT module included in the FSL software (Smith et al., 2004 [77]). For each individual, images were corrected for movement and co-registered to a common stereotaxic space (Montreal Neurological Institute (MNI) template). To minimise unwanted movement-related effects, individuals with an estimated maximum absolute movement > 3.0 mm or an average absolute movement > 0.3 mm were excluded from the study. Normalised volumes were spatially smoothed using the Gaussian filter with a full-width at a half-maximum of 5 mm, and general linear models (GLMs) were fitted to generate individual activation maps for three different contrasts: 1-back vs. baseline, 2-back vs. baseline and 2-back vs. 1-back. Additionally, to control the movement parameters, the movement variables were added to the model as nuisance variables.

Statistical tests were performed at the cluster level with a corrected *p*-value of 0.05 and a z-threshold of 2.3 (using the Standard Field Theory correction implemented in FSL).

Second-level analyses were whole-brain corrected and were performed in all the levels of the task (1-back vs. baseline, 2-back vs. baseline and 2-back vs. 1-back). However, for the interaction analyses (cannabis × *CNR1/CNR2*), we focused on the 2-back vs. 1-back contrast to specifically assess working memory functional response [72], while the other comparisons helped us to interpret the significance of the association.

First, we tested the effect of cannabis (CNU vs. CU) using a regression model that compared brain activity between both groups. Then, the interaction effect of cannabis (CNU and CU) per genotype (*CNR1* or *CNR2*) was investigated using a regression model that assessed whether the slope between groups and genotypes differed. All the regressions were adjusted for age, sex, premorbid-IQ and antipsychotic doses estimated with chlorpromazine equivalents (in mg/day).

Since homozygous for the minor alleles were present at low frequency (n < 5), all analyses were conducted under the dominant model with the SNPs dichotomised (*CNR1*: CC vs. T carriers; *CNR2*: AA vs. G carriers).

To interpret the direction of the interaction results, we estimated individual mean activity scores from the areas where a significant interaction was detected using the FSLSTATS tool in FSL, and afterwards, these values were plotted using SPSS.

#### 4.5.3. Behavioural Performance Association Analyses

The effect of cannabis (CNU vs. CU), as well as the group × genotype interaction on the n-back behavioural scores, were tested for the two task levels (d′1 and d′2) as well as for their difference (d′2-d′1). These analyses were performed through linear regressions adjusted for age, sex and antipsychotic doses as implemented in SPSS.

## 5. Conclusions

Although data from this pilot study should be replicated in larger samples, our findings suggest the role of the genetic make-up as a modulator of the functional integrity of the brain in response to a working memory task in the presence of cannabis use and a first episode of psychosis. Therefore, our study points towards the interest of a better characterisation of the genetic and environmental interplay in the understanding of the heterogeneous outcomes of psychotic disorders in order to develop personalised prevention and therapeutic strategies.

## Figures and Tables

**Table 1:** Sociodemographic and neuropsychological data of the first-episode of psychosis participants included in the study. They were divided into cannabis non-users (CNU) and cannabis users (CU) groups. N = sample size. Premorbid IQ = Premorbid Intelligence Quotient. TAP = Word Accentuation Test. PANSS = Positive and Negative Symptoms Scale. GAF = Global Assessment of Functioning. CPZ = Chlorpromazine. The mean value (standard deviation) is given for the quantitative variables. For statistical comparisons, the *t*-student test was used for the quantitative variables and the chi-squared test for the sex variable.

	Cannabis Non-Users (CNU)	Cannabis Users (CU)	CNU vs. CU Comparison *p*-Value
N	20	20	-
Age (years)	26.19 (7.72)	25.94 (5.31)	0.87
Sex (male/female)	15/5	16/4	0.71
Premorbid IQ (TAP ^a^)	97.24 (11.29)	98.78 (7.95)	0.64
PANSS scale			
PANSS positive	15.90 (5.86)	18.45 (4.80)	0.14
PANSS negative	16.20 (8.46)	16.95 (8.34)	0.78
PANSS general	30.25 (7.60)	33.85 (8.92)	0.18
GAF ^b^	52.11 (11.11)	48.74 (10.94)	0.36
CPZ ^c^ equivalents (mg/day)	292.65 (145.16)	301.20 (181.88)	0.64

^a^ Data of TAP were available for 35 patients. ^b^ Data of GAF were available for 37 patients. ^c^ All patients except 2 were on antipsychotic treatment when the functional Magnetic Resonance Imaging (fMRI) protocol was performed.

**Table 2:** The genotypic distribution of cannabinoid receptor type 1 and 2 genes (*CNR1* and *CNR2***)** between cannabis non-users (CNU) and cannabis users (CU). The genotypes are presented as a two-level variable according to the dominant model used for the analyses. No genotype distribution differences were observed between CNU and CU groups.

Gene (Polymorphism)	Genotypes	Cannabis Non-Users (CNU)	Cannabis Users (CU)	CNU vs. CU Comparison χ^2^ Test, *p*-Value
*CNR1* (rs1049353)	CC	14 (70%)	12 (60%)	0.440, 0.741
	Tcar	6 (30%)	8 (40%)	
*CNR2* (rs2501431)	AA	6 (30%)	9 (45%)	0.960, 0.257
	Gcar	14 (70%)	11 (55%)	

## Data Availability

Data available from the corresponding authors upon reasonable request.

## References

[1] Hilker R., Helenius D., Fagerlund B., Skytthe A., Christensen K., Werge T.M., Nordentoft M., Glenthøj B. (2018). Heritability of Schizophrenia and Schizophrenia Spectrum Based on the Nationwide Danish Twin Register. Biol. Psychiatry.

[2] Sullivan P.F., Kendler K.S., Neale M.C. (2003). Schizophrenia as a Complex Trait Evidence From a Meta-analysis of Twin Studies. Arch. Gen. Psychiatry.

[3] The International Schizophrenia Consortium (2009). Common polygenic variation contributes to risk of schizophrenia and bipolar disorder. Nature.

[4] Trubetskoy V., Pardiñas A.F., Qi T., Panagiotaropoulou G., Awasthi S., Bigdeli T.B., Bryois J., Chen C.-Y., Dennison C.A., Hall L.S. (2022). Mapping genomic loci implicates genes and synaptic biology in schizophrenia. Nature.

[5] Cross-Disorder Group of the Psychiatric Genomics Consortium (2013). Genetic relationship between five psychiatric disorders estimated from genome-wide SNPs. Nat. Genet..

[6] Laurens K.R., Luo L., Matheson S.L., Carr V.J., Raudino A., Harris F., Green M.J. (2015). Common or distinct pathways to psychosis? A systematic review of evidence from prospective studies for developmental risk factors and antecedents of the schizophrenia spectrum disorders and affective psychoses. BMC Psychiatry.

[7] Tost H., Meyer-Lindenberg A. (2012). Puzzling over schizophrenia: Schizophrenia, social environment and the brain. Nat. Med..

[8] Häfner H. (2019). From Onset and Prodromal Stage to a Life-Long Course of Schizophrenia and Its Symptom Dimensions: How Sex, Age, and Other Risk Factors Influence Incidence and Course of Illness. Psychiatry J..

[9] Murray R., Englund A., Abi-Dargham A., Lewis D., Di Forti M., Davies C., Sherif M., McGuire P., D’Souza D. (2017). Cannabis-associated psychosis: Neural substrate and clinical impact. Neuropharmacology.

[10] Marconi A., Di Forti M., Lewis C., Murray R., Vassos E. (2016). Meta-analysis of the Association between the Level of Cannabis Use and Risk of Psychosis. Schizophr. Bull..

[11] Di Forti M., Sallis H., Allegri F., Trotta A., Ferraro L., Stilo S.A., Marconi A., La Cascia C., Marques T.R., Pariante C. (2013). Daily Use, Especially of High-Potency Cannabis, Drives the Earlier Onset of Psychosis in Cannabis Users. Schizophr. Bull..

[12] González-Pinto A., Alberich S., Barbeito S., Gutierrez M., Vega P., Ibáñez B., Haidar M.K., Vieta E., Arango C. (2011). Cannabis and First-Episode Psychosis: Different Long-term Outcomes Depending on Continued or Discontinued Use. Schizophr. Bull..

[13] Mazzoncini R., Donoghue K., Hart J., Morgan C., Doody G.A., Dazzan P., Jones P.B., Morgan K., Murray R.M., Fearon P. (2010). Illicit substance use and its correlates in first episode psychosis. Acta Psychiatr. Scand..

[14] Marino L., Scodes J., Richkin T., Alves-Bradford J.-M., Nossel I., Wall M., Dixon L. (2020). Persistent cannabis use among young adults with early psychosis receiving coordinated specialty care in the United States. Schizophr. Res..

[15] Pardo M., Matalí J.L., Sivoli J., Regina V.-B., Butjosa A., Dolz M., Sánchez B., Barajas A., Del Cacho N., Baños I. (2021). Early onset psychosis and cannabis use: Prevalence, clinical presentation and influence of daily use. Asian J. Psychiatry.

[16] Melis M., Muntoni A.L.M., Pistis M.M. (2012). Endocannabinoids and the Processing of Value-Related Signals. Front. Pharmacol..

[17] Herkenham M., Lynn A.B., Little M.D., Johnson M.R., Melvin L.S., De Costa B.R., Rice K.C. (1990). Cannabinoid receptor localization in brain. Proc. Natl. Acad. Sci USA.

[18] Fernández-Ruiz J., Hernández M.L., Ramos J.A. (2010). Cannabinoid-Dopamine Interaction in the Pathophysiology and Treatment of CNS Disorders. CNS Neurosci. Ther..

[19] Melis M., Pistis M., Perra S., Muntoni A.L., Pillolla G., Gessa G.L. (2004). Endocannabinoids Mediate Presynaptic Inhibition of Glutamatergic Transmission in Rat Ventral Tegmental Area Dopamine Neurons through Activation of CB1 Receptors. J. Neurosci..

[20] Onaivi E.S., Ishiguro H., Gong J.P., Patel S., Perchuk A., Meozzi P.A., Myers L., Mora Z., Tagliaferro P., Gardner E. (2006). Discovery of the Presence and Functional Expression of Cannabinoid CB2 Receptors in Brain. Ann. N. Y. Acad. Sci..

[21] Liu Q.-R., Pan C.-H., Hishimoto A., Li C.-Y., Xi Z.-X., Llorente-Berzal A., Viveros M.-P., Ishiguro H., Arinami T., Onaivi E.S. (2009). Species differences in cannabinoid receptor 2 (*CNR2*gene): Identification of novel human and rodent CB2 isoforms, differential tissue expression and regulation by cannabinoid receptor ligands. Genes Brain Behav..

[22] Jordan C.J., Xi Z.-X. (2019). Progress in brain cannabinoid CB2 receptor research: From genes to behavior. Neurosci. Biobehav. Rev..

[23] Chen D.-J., Gao M., Gao F.-F., Su Q.-X., Wu J. (2017). Brain cannabinoid receptor 2: Expression, function and modulation. Acta Pharmacol. Sin..

[24] Zhang H., Bi G.-H., Li X., Li J., Qu H., Zhang S.-J., Li C.-Y., Onaivi E.S., Gardner E.L., Xi Z.-X. (2014). Species Differences in Cannabinoid Receptor 2 and Receptor Responses to Cocaine Self-Administration in Mice and Rats. Neuropsychopharmacology.

[25] Schroll H., Vitay J., Hamker F.H. (2012). Working memory and response selection: A computational account of interactions among cortico-basalganglio-thalamic loops. Neural Netw..

[26] Lee J., Park S. (2005). Working Memory Impairments in Schizophrenia: A Meta-Analysis. J. Abnorm. Psychol..

[27] Mesholam-Gately R.I., Giuliano A.J., Goff K.P., Faraone S.V., Seidman L.J. (2009). Neurocognition in first-episode schizophrenia: A meta-analytic review. Neuropsychology.

[28] Zhornitsky S., Pelletier J., Assaf R., Giroux S., Li C.-S.R., Potvin S. (2021). Acute effects of partial CB1 receptor agonists on cognition—A meta-analysis of human studies. Prog. Neuro-Psychopharmacol. Biol. Psychiatry.

[29] Jacobsen L.K., Mencl W.E., Westerveld M., Pugh K.R. (2004). Impact of Cannabis Use on Brain Function in Adolescents. Ann. N. Y. Acad. Sci..

[30] Harvey M.A., Sellman J.D., Porter R.J., Frampton C.M. (2007). The relationship between non-acute adolescent cannabis use and cognition. Drug Alcohol Rev..

[31] Schoeler T., Petros N., Di Forti M., Klamerus E., Foglia E., Ajnakina O., Gayer-Anderson C., Colizzi M., Quattrone D., Behlke I. (2016). Effects of continuation, frequency, and type of cannabis use on relapse in the first 2 years after onset of psychosis: An observational study. Lancet Psychiatry.

[32] Bogaty S.E., Lee R.S., Hickie I.B., Hermens D.F. (2018). Meta-analysis of neurocognition in young psychosis patients with current cannabis use. J. Psychiatr. Res..

[33] Løberg E.-M. (2009). Cannabis use and cognition in schizophrenia. Front. Hum. Neurosci..

[34] Yucel M., Bora E., Lubman D.I., Solowij N., Brewer W.J., Cotton S.M., Conus P., Takagi M.J., Fornito A., Wood S.J. (2012). The Impact of Cannabis Use on Cognitive Functioning in Patients with Schizophrenia: A Meta-analysis of Existing Findings and New Data in a First-Episode Sample. Schizophr. Bull..

[35] Mata I., Rodríguez-Sánchez J.M., Pelayo-Terán J.M., Pérez-Iglesias R., González-Blanch C., Ramírez-Bonilla M., Martínez-García O., Vázquez-Barquero J.L., Crespo-Facorro B. (2008). Cannabis abuse is associated with decision-making impairment among first-episode patients with schizophrenia-spectrum psychosis. Psychol. Med..

[36] Kanayama G., Rogowska J., Pope H.G., Gruber S.A., Yurgelun-Todd D.A. (2004). Spatial working memory in heavy cannabis users: A functional magnetic resonance imaging study. Psychopharmacology.

[37] Smith A.M., Longo C.A., Fried P.A., Hogan M.J., Cameron I. (2010). Effects of marijuana on visuospatial working memory: An fMRI study in young adults. Psychopharmacology.

[38] Schweinsburg A.D., Schweinsburg B.C., Medina K.L., McQueeny T., Brown S.A., Tapert S.F. (2010). The Influence of Recency of Use on fMRI Response During Spatial Working Memory in Adolescent Marijuana Users. J. Psychoact. Drugs.

[39] Jager G., Block R.I., Luijten M., Ramsey N.F. (2010). Cannabis Use and Memory Brain Function in Adolescent Boys: A Cross-Sectional Multicenter Functional Magnetic Resonance Imaging Study. J. Am. Acad. Child Adolesc. Psychiatry.

[40] Potvin S., Bourque J., Durand M., Lipp O., Lalonde P., Stip E., Grignon S., Mendrek A. (2013). The Neural Correlates of Mental Rotation Abilities in Cannabis-Abusing Patients with Schizophrenia: An fMRI Study. Schizophr. Res. Treat..

[41] Bourque J., Mendrek A., Durand M., Lakis N., Lipp O., Stip E., Lalonde P., Grignon S., Potvin S. (2013). Cannabis abuse is associated with better emotional memory in schizophrenia: A functional magnetic resonance imaging study. Psychiatry Res..

[42] Løberg E.-M., Nygård M., Berle J., Johnsen E., Kroken R.A., Jørgensen H.A., Hugdahl K. (2012). An fMRI Study of Neuronal Activation in Schizophrenia Patients with and without Previous Cannabis Use. Front. Psychiatry.

[43] Cunha P.J., Rosa P.G.P., Ayres A.D.M., Duran F.L., Santos L.C., Scazufca M., Menezes P.R., dos Santos B., Murray R.M., Crippa J.A.S. (2013). Cannabis use, cognition and brain structure in first-episode psychosis. Schizophr. Res..

[44] Pelayo-Teran J.M., Suarez-Pinilla P., Chadi N., Crespo-Facorro B. (2012). Gene-Environment Interactions Underlying the Effect of Cannabis in First Episode Psychosis. Curr. Pharm. Des..

[45] Bioque M., Mas S., Costanzo M.C., Cabrera B., Lobo A., González-Pinto A., Rodriguez-Toscano E., Corripio I., Vieta E., Baeza I. (2019). Gene-environment interaction between an endocannabinoid system genetic polymorphism and cannabis use in first episode of psychosis. Eur. Neuropsychopharmacol..

[46] Ujike H., Takaki M., Nakata K., Tanaka Y., Takeda T., Kodama M., Fujiwara Y., Sakai A., Kuroda S. (2002). CNR1, central cannabinoid receptor gene, associated with susceptibility to hebephrenic schizophrenia. Mol. Psychiatry.

[47] Martínez-Gras I., Hoenicka J., Ponce G., Rodríguez-Jiménez R., Jiménez-Arriero M.A., Perez-Hernandez E., Ampuero I., Ramos-Atance J.A., Palomo T., Rubio G. (2006). (AAT)n repeat in the cannabinoid receptor gene, CNR1: Association with schizophrenia in a Spanish population. Eur. Arch. Psychiatry Clin. Neurosci..

[48] Ho B.-C., Wassink T.H., Ziebell S., Andreasen N.C. (2011). Cannabinoid receptor 1 gene polymorphisms and marijuana misuse interactions on white matter and cognitive deficits in schizophrenia. Schizophr. Res..

[49] Kuzman M.R., Kuharic D.B., Ganoci L., Makaric P., Kekin I., Gajsak L.R., Prpic N., Bozina T., Bajić Z., Bozina N. (2019). Association of CNR1 genotypes with changes in neurocognitive performance after eighteen-month treatment in patients with first-episode psychosis. Eur. Psychiatry.

[50] Suárez-Pinilla P., Roiz-Santiañez R., de la Foz V.O.-G., Guest P.C., Ayesa-Arriola R., Córdova-Palomera A., Tordesillas-Gutierrez D., Crespo-Facorro B. (2015). Brain structural and clinical changes after first episode psychosis: Focus on cannabinoid receptor 1 polymorphisms. Psychiatry Res..

[51] Gouvêa E.S., Filho A.F.S., Ota V.K., Mrad V., Gadelha A., Bressan R.A., Cordeiro Q., Belangero S.I. (2017). The role of the CNR1 gene in schizophrenia: A systematic review including unpublished data. Revista Brasileira de Psiquiatria.

[52] Onwuameze O.E., Nam K.W., Epping E.A., Wassink T.H., Ziebell S., Andreasen N.C., Ho B.-C. (2013). *MAPK14* and *CNR1* gene variant interactions: Effects on brain volume deficits in schizophrenia patients with marijuana misuse. Psychol. Med..

[53] Schacht J.P., Hutchison K.E., Filbey F.M. (2012). Associations between Cannabinoid Receptor-1 (CNR1) Variation and Hippocampus and Amygdala Volumes in Heavy Cannabis Users. Neuropsychopharmacology.

[54] Colizzi M., Iyegbe C., Powell J., Ursini G., Porcelli A., Bonvino A., Taurisano P., Romano R., Masellis R., Blasi G. (2015). Interaction between Functional Genetic Variation of DRD2 and Cannabis Use on Risk of Psychosis. Schizophr. Bull..

[55] Taurisano P., Antonucci L.A., Fazio L., Rampino A., Romano R., Porcelli A., Masellis R., Colizzi M., Quarto T., Torretta S. (2016). Prefrontal activity during working memory is modulated by the interaction of variation in CB1 and COX2 coding genes and correlates with frequency of cannabis use. Cortex.

[56] Taurisano P., Pergola G., Monda A., Antonucci L.A., Di Carlo P., Piarulli F., Passiatore R., Papalino M., Romano R., Monaco A. (2021). The interaction between cannabis use and a CB1-related polygenic co-expression index modulates dorsolateral prefrontal activity during working memory processing. Brain Imaging Behav..

[57] Quickfall J., Crockford D. (2006). Brain Neuroimaging in Cannabis Use: A Review. J. Neuropsychiatry Clin. Neurosci..

[58] Haber S.N., Knutson B. (2010). The Reward Circuit: Linking Primate Anatomy and Human Imaging. Neuropsychopharmacology.

[59] Oldham S., Murawski C., Fornito A., Youssef G., Yücel M., Lorenzetti V. (2018). The anticipation and outcome phases of reward and loss processing: A neuroimaging meta-analysis of the monetary incentive delay task. Hum. Brain Mapp..

[60] Curran H.V., Freeman T., Mokrysz C., Lewis D., Morgan C.J.A., Parsons L.H. (2016). Keep off the grass? Cannabis, cognition and addiction. Nat. Rev. Neurosci..

[61] Ishiguro H., Horiuchi Y., Ishikawa M., Koga M., Imai K., Suzuki Y., Morikawa M., Inada T., Watanabe Y., Takahashi M. (2010). Brain Cannabinoid CB2 Receptor in Schizophrenia. Biol. Psychiatry.

[62] Coleman J.R., Madularu D., Ortiz R.J., Athanassiou M., Knudsen A., Alkislar I., Cai X., Kulkarni P.P., Cushing B.S., Ferris C.F. (2022). Changes in brain structure and function following chronic exposure to inhaled vaporised cannabis during periadolescence in female and male mice: A multimodal MRI study. Addict. Biol..

[63] Spindle T.R., Kuwabara H., Eversole A., Nandi A., Vandrey R., Antoine D.G., Umbricht A., Guarda A.S., Wong D.F., Weerts E.M. (2021). Brain imaging of cannabinoid type I (CB _1_) receptors in women with cannabis use disorder and male and female healthy controls. Addict. Biol..

[64] Basavarajappa B.S., Subbanna S. (2022). Molecular Insights into Epigenetics and Cannabinoid Receptors. Biomolecules.

[65] Tao R., Li C., Jaffe A.E., Shin J.H., Deep-Soboslay A., Yamin R., Weinberger D.R., Hyde T.M., Kleinman J.E. (2020). Cannabinoid receptor CNR1 expression and DNA methylation in human prefrontal cortex, hippocampus and caudate in brain development and schizophrenia. Transl. Psychiatry.

[66] Kebir O., Chaumette B., Rivollier F., Miozzo F., Perreault L.P.L., Barhdadi A., Provost S., Plaze M., Bourgin J., the ICAAR team (2017). Methylomic changes during conversion to psychosis. Mol. Psychiatry.

[67] Kay S.R., Flszbeln A., Qpjer L.A. (1987). The Positive and Negative Syndrome Scale (PANSS) for Schizophrenia. Schizophr. Bull..

[68] Wallwork R., Fortgang R., Hashimoto R., Weinberger D., Dickinson D. (2012). Searching for a consensus five-factor model of the Positive and Negative Syndrome Scale for schizophrenia. Schizophr. Res..

[69] Gomar J.J., Ortiz-Gil J., McKenna P.J., Salvador R., Sans-Sansa B., Sarró S., Guerrero A., Pomarol-Clotet E. (2011). Validation of the Word Accentuation Test (TAP) as a means of estimating premorbid IQ in Spanish speakers. Schizophr. Res..

[70] Gevins A., Cutillo B. (1993). Spatiotemporal dynamics of component processes in human working memory. Electroencephalogr. Clin. Neurophysiol..

[71] Green D.M., Swets J.A. (1966). Signal Detection Theory and Psychophysics.

[72] Egli T., Coynel D., Spalek K., Fastenrath M., Freytag V., Heck A., Loos E., Auschra B., Papassotiropoulos A., De Quervain D.J.-F. (2018). Identification of Two Distinct Working Memory-Related Brain Networks in Healthy Young Adults. eNeuro.

[73] Hill S.Y., Sharma V., Jones B.L. (2016). Lifetime use of cannabis from longitudinal assessments, cannabinoid receptor (CNR1) variation, and reduced volume of the right anterior cingulate. Psychiatry Res. Neuroimaging.

[74] Costa M., Squassina A., Congiu D., Chillotti C., Niola P., Galderisi S., Pistis M., Del Zompo M. (2013). Investigation of endocannabinoid system genes suggests association between peroxisome proliferator activator receptor-α gene (PPARA) and schizophrenia. Eur. Neuropsychopharmacol..

[75] Hamdani N., Tabeze J.-P., Ramoz N., Ades J., Hamon M., Sarfati Y., Boni C., Gorwood P. (2008). The CNR1 gene as a pharmacogenetic factor for antipsychotics rather than a susceptibility gene for schizophrenia. Eur. Neuropsychopharmacol..

[76] Gerra M.C., Jayanthi S., Manfredini M., Walther D., Schroeder J., Phillips K.A., Cadet J.L., Donnini C. (2018). Gene variants and educational attainment in cannabis use: Mediating role of DNA methylation. Transl. Psychiatry.

[77] Smith S.M., Jenkinson M., Woolrich M.W., Beckmann C.F., Behrens T.E.J., Johansen-Berg H., Bannister P.R., De Luca M., Drobnjak I., Flitney D.E. (2004). Advances in functional and structural MR image analysis and implementation as FSL. Neuroimage.

